# “Scan-(pre)Plan-Treat” Workflow for Bone Metastases Using the Ethos Therapy System: A Single-Center, In Silico Experience

**DOI:** 10.1016/j.adro.2023.101258

**Published:** 2023-04-24

**Authors:** Eva Oldenburger, Robin De Roover, Kenneth Poels, Tom Depuydt, Sofie Isebaert, Karin Haustermans

**Affiliations:** a Department of Radiation Oncology, University Hospitals Leuven, Leuven, Belgium; b Department of Oncology, KU Leuven, Leuven, Belgium; c Department of Palliative Care, University Hospitals Leuven, Leuven, Belgium

## Abstract

**Purpose:**

To report on the accuracy of automated delineation, treatment plan quality, and duration of an in-silico “scan-(pre)plan-treat” (SPT) workflow for vertebral bone metastases using a 1 × 8 Gy regimen.

**Method and Materials:**

The cloud-based emulator system of the Ethos therapy system was used to adapt an organ-at-risk-sparing preplan created on the diagnostic CT to the anatomy-of-the-day using the cone beam CT made before treatment.

**Results:**

SPT using the Ethos emulator system resulted in relatively good coverage of the PTV and acceptable dose to the OAR. Delivery time and plan homogeneity was the best for 7-field IMRT plan template.

**Conclusions:**

A SPT workflow formula results in a highly conformal treatment delivery while maintaining an acceptable timeframe for the patient on the treatment couch.

## Introduction

**Figure 1:**
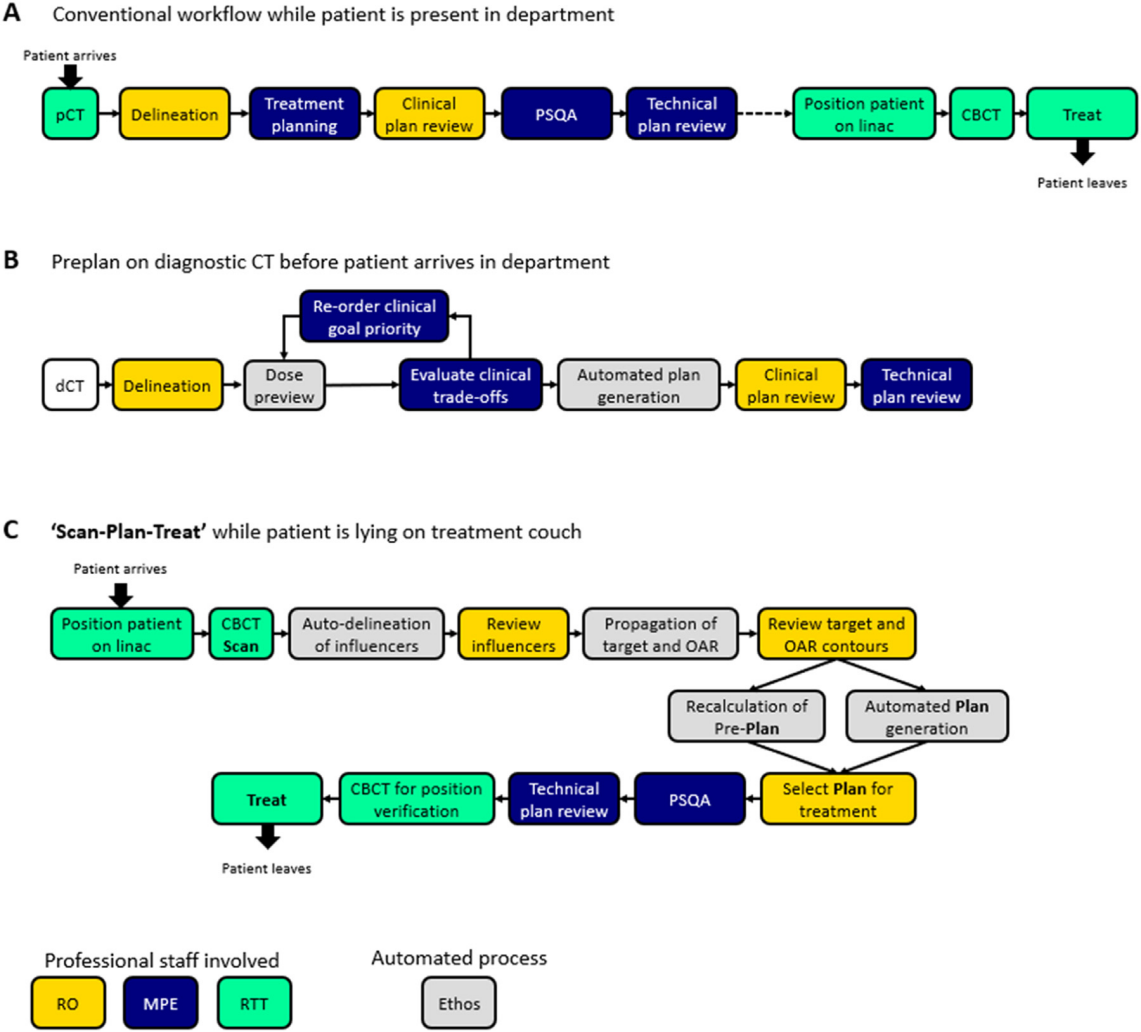
Schematic overview of the different workflow steps performed in (A) the conventional workflow while the patient is present in the department, (B) the preplan on diagnostic computed tomography before the patient arrives in the department, and (C) the scan-plan-treat formula performed on cone beam computed tomography while the patient is lying on the treatment couch. The radiation oncology professionals involved have been denoted for each task, together with the automated processes performed by the Ethos therapy adaptive workflow.

Painful bone metastases are a common indication for palliative radiotherapy (RT), with 1 × 8 Gy being the most recommended treatment schedule.^1^^,^^2^ After referral, patients usually have to wait a few days for treatment to start. This time is necessary for thorough patient assessment, review of his/her diagnostic imaging and to set the treatment indication. When a patient arrives in the department of radiation-oncology for treatment, first a planning CT scan in treatment position (pCT) is performed. Subsequently, the patient needs to wait while the delineation of the target volume and organs at risk (OAR), dose prescription, treatment planning, plan review and patient-specific quality assurance (PSQA) are performed by different professionals. After this treatment preparation process, the treatment can finally be delivered (Fig. 1A). Even in case of an emergency, patients may have to wait several hours for treatment to start, as this preparation process is necessary for every treatment.^3^

Until recently, speeding up this process was difficult, due to technical and logistical challenges. However, the development of integrated online adaptive radiotherapy (oART) solutions, such as the Ethos therapy system (Varian Medical Systems, Palo Alto, CA) which combines artificial intelligence (AI)-based auto-segmentation and automated treatment planning methods into a single treatment platform, has enabled to perform online adaptive treatments in a limited timeframe.^4^^,^^5^

In this study we used a cloud-based emulator system that mimics the Ethos therapy system's adaptive workflow to perform a so-called “scan-(pre)plan-treat” (SPT) workflow. Here, we report about the feasibility and in-silico results concerning the accuracy of automated delineation, treatment plan quality, and duration of this SPT workflow to vertebral bone metastases for a 1 × 8 Gy regimen.

## Methods and Materials

This study was approved by our ethics committee research board. The authors confirm that written informed consent was obtained from the involved patients or, if appropriate, from the parent, guardian, or power of attorney of the involved patient(s), and they have given approval for this information to be published in this article.

The data set was made up of 7 patients. These patients were selected based on the availability of high-quality diagnostic CT (dCT), pCT, and cone beam CT (CBCT) images. Our data set resulted in emulated treatments to 18 vertebral localizations.

For all localizations, delineation and treatment planning were performed on the dCT by an experienced radiation-oncologist and physicist, respectively, based on our department's criteria (Table 1). Clinical target volume (CTV) included the entire vertebra; planning target volume (PTV) was derived from the CTV by adding a 1-cm isotropic margin. Relevant OARs were determined by treatment localization.

**Table 1:** Dose constraints for palliative RT treatments as used in our department

Structure	Dose constraint
Target	
CTV	D_99%_ ≥ 95% prescribed dose
PTV	D_99%_ ≥ 90% prescribed dose
	D_0.03cc_ ≤ 107% prescribed dose
Organ at risk	
Spinal canal	D_0.03cc_ ≤ 105% prescribed dose
Heart	D_0.03cc_ ≤ 105% prescribed dose
	D_mean_ ≤ 20 Gy
Esophagus	D_0.03cc_ ≤ 100% prescribed dose
Lungs	D_mean_ ≤ 18 Gy
Bowel	D_0.03cc_ ≤ 105% prescribed dose
Kidney (each)	D_mean_ ≤ 12 Gy

*Abbreviations:* CTV = clinical target volume; PTV = planning target volume; RT = radiation therapy.

These constraints are valid for prescribed doses of 1 × 8 and 5 × 4 Gy.

The delineation and planning were performed without any time constraints to generate the best plan possible on the dCT, called the “preplan.” For this, we used the Ethos Intelligent Optimization Engine (IOE) for automated plan generation based on a prioritized list of clinical goals (Fig. 1B). These clinical goals have the dual function of evaluating the target coverage and OAR dose and steering the plan's optimization process via the IOE that converts the clinical goals into optimization objectives. Primary target goal was PTV covering D_99.0%_ ≥ 92.5%; secondary goals were PTV covering D_95.0%_ ≥ 95.0%, PTV D_0.03cc_ < 8.56 Gy, and CTV D_99.0%_ ≥ 99.0%. OARs considered for plan optimization were the cauda equina/spinal canal (D_0.03cc_ < 8.56 Gy) and each kidney (D_mean_ < 12 Gy), following department's guidelines (Table 1). All other OARs, such as stomach, bowel bag, lungs, and heart, were contoured but not used to steer plan optimization.

The IOE generated 5 treatment plans automatically using standard templates: 7-, 9-, and 12-beam intensity modulated RT (IMRT) and 2- and 3-arc volumetric modulated arc therapy (VMAT). Plan quality (PTV coverage, homogeneity, and OAR dose) and the plan generation time of each template were evaluated and compared. A short plan generation time was considered essential to keep the duration on the treatment couch while performing online adaptive RT acceptable for patients. Dose delivery time was recorded for all 5 templates for 3 cases by performing a dummy delivery of the preplans on Halcyon.

In an SPT workflow, the patient would come to the hospital on the treatment day to be positioned in a comfortable, stable position on the treatment couch, after which a CBCT will be acquired (Fig. 1C). To verify the position of both target and OARs, the Ethos adaptive workflow uses artificial intelligence–based automatically delineated “influencer” structures. Influencer structures are structures that are in the closest proximity to the target and have the biggest effect on the shape of common clinical target structures. These structures are used to perform a structure-guided deformable image registration between dCT and CBCT. In turn, this deformable image registration is used to propagate structures that are not automatically delineated (such as the CTV) from the dCT to the CBCT and to generate a synthetic CT (sCT) by deforming the dCT into the CBCT geometry. The influencer structures and propagated contours are reviewed by a radiation oncologist (Fig. 1C). Using the reviewed contours together with the sCT, an adapted plan is generated by IOE using the same list of clinical goals as for the preplan. Simultaneously, the preplan is rigidly registered to the CBCT and the dose is recalculated on the sCT to evaluate the delivered dose if the patient would be realigned by a couch correction and treated with the nonadapted preplan. Based on the dose distribution on sCT of both plans, the radiation oncologist selects the best plan (either the nonadapted, propagated plan on the CBCT or the adapted plan) for treatment, after which patient-specific quality assurance and technical plan review are performed (Fig. 1C). A second CBCT can be acquired to verify the patient position and to correct potential movement during the workflow via couch correction if necessary. The quality of the different imaging modalities is shown in Fig. E1.

To evaluate the accuracy of the proposed workflow, the target and OAR contours on each CBCT were delineated offline. These contours were used as ground truth object delineations to re-evaluate the target coverage and OAR dose (offline evaluation) and to compare these to the values obtained during the SPT workflow (online evaluation). The number of corrections of the auto-delineated contours during the adaptive workflow was recorded together with the time required to perform this review. The automated planning time during the workflow and the total time of the adaptive workflow from CBCT acquisition until selection of the plan used for treatment was also recorded.

## Results

All techniques resulted in good PTV coverage and acceptable dose to the OARs. However, VMAT plans were less homogeneous, with PTV D_0.03cc_ exceeding the constraint for most cases, whereas all but 1 IMRT plan were within the constraints (Fig. E2).

**Figure 2:**
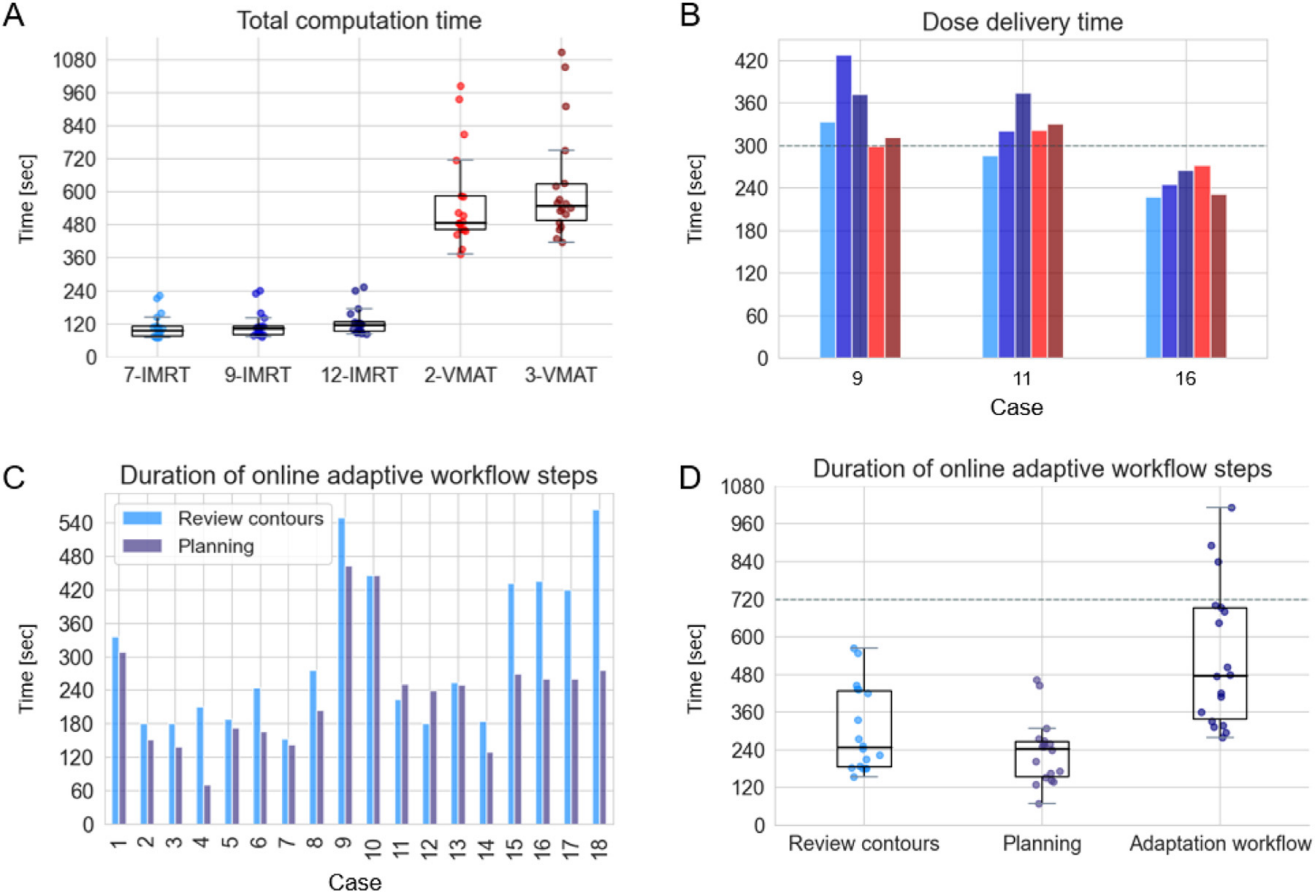
Duration of various steps in the scan-(pre)plan-treat workflow. (A) Total computation time of the Intelligent Optimization Engine to generate the preplan for the 5 different standard plan templates. (B) Dose delivery time of the 5 standard plan templates as measured during a dummy delivery on Halcyon. (C) Duration of the different steps in the scan-plan-treat online adaptive workflow for each case individually and (D) for all cases, combined in a boxplot. The duration of a typical treatment session of 12 minutes is denoted in (D) by a dashed line for comparison. Note that the time required for patient positioning, cone beam computed tomography acquisition, and treatment delivery is not included in the adaptation workflow in (D).

The computation time of the IOE to generate IMRT plans was significantly shorter than that for VMAT plans: median 1 minute 43 seconds versus median 8 minutes 47 seconds, respectively (Fig. 2A). Plan delivery time was shortest for 7-beam IMRT plans, ranging from 3 minutes 47 seconds to 5 minutes 33 seconds (Fig. 2B). Therefore, it was decided to use the 7-field IMRT plan template for the in silico evaluation of the SPT workflow.

The spinal canal influencer structure needed correction in 8/18 cases, mostly expansion to the entire spinal canal. The propagated CTV contour needed correction in 15/18 cases because of overlap with adjecent vertebra or incomplete contouring of processes. The PTV expansion was created automatically after contour approval. The OARs needing the most modification were the kidneys and bowel bag.

The autosegmentation, with review and correction of the influencer structures and propagated contours, took median 4 minutes 8 seconds, starting from the CBCT acquisition. Automated planning and dose recalculation were finished in median 4 minutes 4 seconds after contour approval. In total, the adaptation workflow had a median duration of 7 minutes 57 seconds, ranging from 4 minutes 40 seconds to 16 minutes 52 seconds after CBCT acquisition (Fig. 2C, 2D).

**Figure 3:**
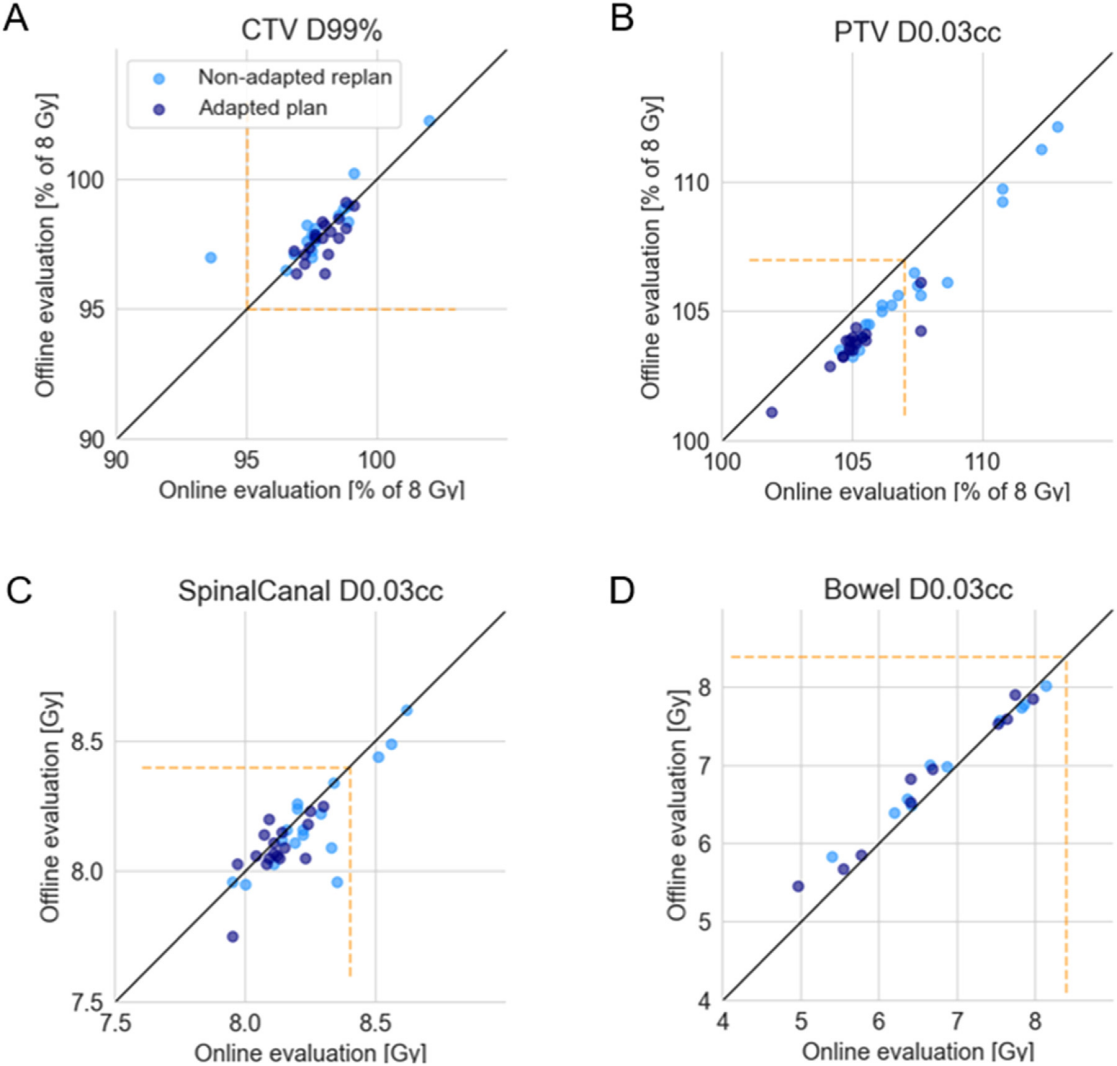
Offline verification of target coverage and organ-at-risk dose evaluated during the online scan-plan-treat workflow for (A) clinical target volume D_99%_, (B) planning target volume D_0.03cc_, (C) spinal canal D_0.03cc_, and (D) bowel D_0.03cc_. The yellow dashed line indicates the dose constraints in Table 1 for each structure. Datapoints above the solid black line indicate an underestimation of the online evaluation for the dose-volume histogram metric, whereas datapoints below the line indicate an overestimation of the dose-volume histogram metric.

There was agreement between the online evaluated and offline evaluated target coverage and OAR doses (Fig. 3). Plans that satisfied the CTV D_99%_ ≥ 95% prescribed dose clinical goal during the SPT workflow also did during offline evaluation. Online evaluation slightly overestimated the PTV D_0.03cc_. Both spinal canal D_0.03cc_ and bowel D_0.03cc_ showed good agreement between the online and offline evaluated values.

## Discussion

Our research explored an SFRT SPT workflow using the Ethos emulator system. We found a planning strategy delivering highly conformal treatment with good coverage and sparing of the OARs with an on-couch time of less than 20 minutes using a 7-field IMRT technique.

Previously, Wong et al^6^ used a single-fraction treatment with a simple 1- or 2-field technique. Although this technique was effective in streamlining delivery, poor conformality resulted in high radiation doses to surrounding organs. Létourneau et al^3^ showed the technical feasibility of CBCT-guided online planning and delivery for palliative treatment to the spine in a treatment slot of less than 30 minutes on a phantom. Nierer et al^7^ used the dCT top plan and the CBCT for setup accuracy, but unfortunately did not report on the time spent on the treatment couch. More recently, Schiff et al^8^ showed the feasibility of simulation-free planning of SFRT schedules but with patients on the treatment couch for 40 minutes. The effect of using the dCT houndsfield units values to generate the sCT for dose calculation was not established in our study, but prior work found that the use of dCT houndsfield units data may be suitable for many cases in an emergency RT setting.^7^ Furthermore, the use of density-calibrated CBCT could allow for direct dose calculation on CBCT, obviating the need for an sCT.^9^

Limitations of our analysis include the small sample size, evaluating exclusively vertebral irradiations, and the use of the emulator system, which may affect the calculation times. On the other hand, this is one of the first studies that looked at an SPT workflow focusing on a limited timeframe and therefore making this workflow clinically feasible. As such, we believe that the results of our analysis can be used in further clinical trials, such as the DART trial (NCT05233904).^10^ This randomized trial will evaluate dCT-planned SFRT against standard pCT-planned RT, with the primary endpoint being the time in center on treatment day, and we aim to also focus on time on the treatment couch.

## Conclusion

This in silico study, using an emulator system, demonstrates that treating vertebral bone metastases with online adaptive IMRT on Ethos through an SPT workflow formula results in highly conformal treatment delivery within an acceptable timeframe for the patient on the treatment couch.

## Disclosures

The authors declare that they have no known competing financial interests or personal relationships that could have appeared to influence the work reported in this paper.

## Appendix Supplementary materials

SPT_AiRO_resubmission_supplementaryfigures_clean Image, application 1

## Footnotes

Supplementary material associated with this article can be found in the online version at doi:10.1016/j.adro.2023.101258.
