# Supplementary material for: “Scan-(pre)Plan-Treat” Workflow for Bone Metastases Using the Ethos Therapy System: A Single-Center, In Silico Experience
Source: Adv Radiat Oncol. 2023 Apr 24;8(6):101258. doi: 10.1016/j.adro.2023.101258 (PMC10248728; doi:10.1016/j.adro.2023.101258)
Supplement: SPT_AiRO_resubmission_supplementaryfigures_clean [file mmc1.docx]

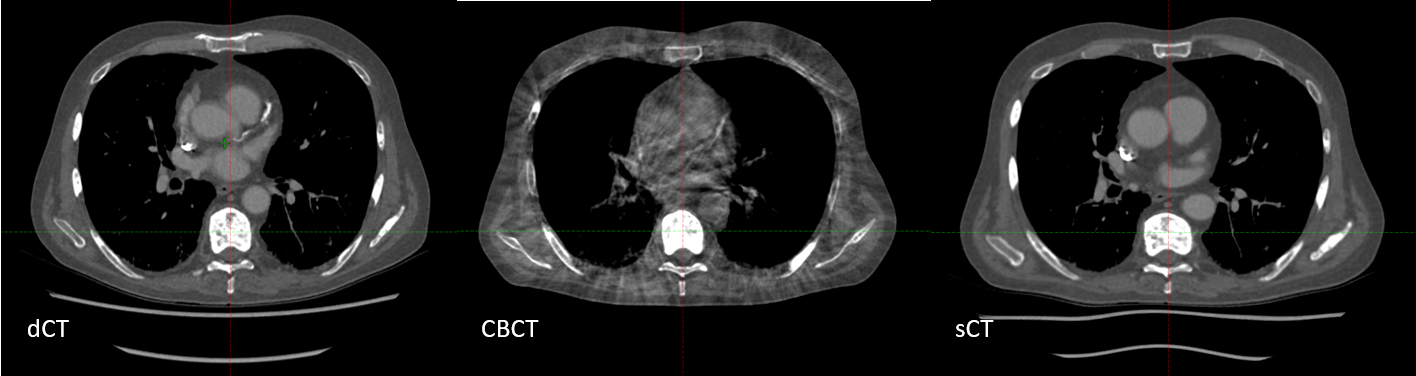


**Supplementary figure 1**: **Vertebra D8 in shown in the different imaging modalities used in the SPT workflow.**Image depicted in window level ‘Bone’ (range: -400 to 800 HU)
dCT: diagnostic CT; CBCT: Cone Beam CT; sCT: synthetic CT


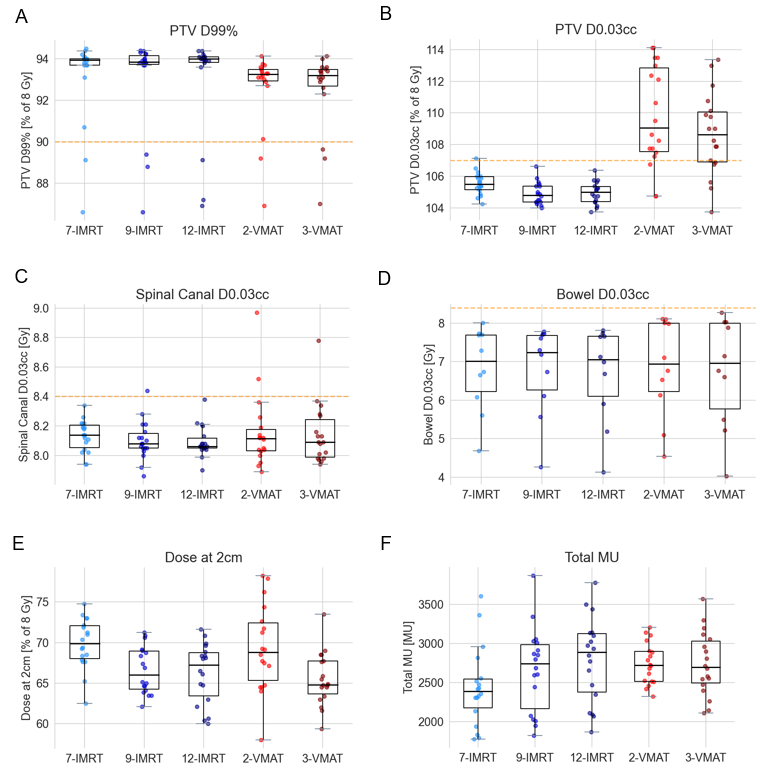


**Supplementary figure 2. Plan quality and characteristic for the five standard plan templates generated by the IOE during preplanning.** (A) PTV D99% target coverage, (B) PTV D0.03cc as surrogate for target homogeneity, (C) Spinal canal D0.03cc and (D) Bowel D0.03cc OAR dose, (E) maximum dose spillage at 2 cm distance from the PTV and (F) total monitor units of the plan. 1 MU is defined as 1 cGy in reference conditions, i.e. at 10 cm depth in water for a 10 x 10 cm² open field at 90 cm source-surface distance. PTV = planning target volume; MU = monitor unit.
